# Sentinel site community surveillance of mortality and nutritional status in southwestern Central African Republic, 2010

**DOI:** 10.1186/1478-7954-10-18

**Published:** 2012-09-04

**Authors:** Grazia M Caleo, Aly Penda Sy, Serge Balandine, Jonathan Polonsky, Pedro Pablo Palma, Rebecca Freeman Grais, Francesco Checchi

**Affiliations:** 1 European Programme for Intervention Epidemiology Training (EPIET), European Centre for Disease Prevention and Control (ECDC), Stockholm, Sweden; 2 Epicentre, 8 rue Saint-Sabin, Paris, France; 3 Médecins Sans Frontières, Operational Centre Barcelona-Athens, Nou de la Rambla, 26, Barcelona, Spain; 4 Health and Nutrition Tracking Service, Geneva, Switzerland; 5 Faculty of Infectious and Tropical Diseases, London School of Hygiene and Tropical Medicine, Keppel Street, London, United Kingdom

**Keywords:** Mortality, Malnutrition, Central African Republic, Surveillance, Rural, Humanitarian

## Abstract

**Background:**

During 2010, a community-based, sentinel site prospective surveillance system measured mortality, acute malnutrition prevalence, and the coverage of a Médecins Sans Frontières (MSF) intervention in four sous-préfectures of Lobaye prefecture in southwestern Central African Republic. We describe this surveillance system and its evaluation.

**Methods:**

Within 24 randomly selected sentinel sites, home visitors performed a census, weekly demographic surveillance of births, deaths, and in- or out-migration, and weekly anthropometry on a sample of children. We evaluated the system through various methods including capture-recapture analysis and repeat census.

**Results:**

The system included 18,081 people at baseline. Over 32 weeks, the crude death rate was 1.0 (95% confidence interval [CI]: 0.8-1.2) deaths per 10,000 person-days (35 deaths per 1,000 person-years), with higher values during the rainy season. The under-5 death rate was approximately double. The prevalence of severe acute malnutrition (SAM) was 3.0% (95% CI: 2.3-4.0), almost half featuring kwashiorkor signs. The coverage of SAM treatment was 29.1%. The system detected >90% of deaths, and >90% of death reports appeared valid. However, demographic surveillance yielded discrepancies with the census and an implausible rate of population growth, while the predictive value of SAM classification was around 60%.

**Discussion:**

We found evidence of a chronic health crisis in this remote region. MSF's intervention coverage improved progressively. Mortality data appeared valid, but inaccuracies in population denominators and anthropometric measurements were noted. Similar systems could be implemented in other remote settings and acute emergencies, but with certain technical improvements.

## Introduction

The Central African Republic (CAR) is a landlocked country in Central Africa with an estimated population of 4.7 million in 2009 [1]. CAR is one of the poorest countries in Africa, with two-thirds of the population living on less than one United States dollar (USD) per day. Health indicators are among the worst globally [2].

Diamond mining is one of the main sources of wealth in the southwestern part of the country. However, since 2009, the economy of this region has been suffering from the effects of the global downturn in demand for diamonds and from restrictions on mining concessions. The convergence of these factors resulted in a crisis in the local economy with an increase of food prices, a decline in agricultural production, and a situation of food insecurity [3].

A survey conducted in the southwestern Carnot prefecture in 2009 found crude and under-5 death rates of 1.2 per 10,000 person-days and 2.3 per 10,000 person-days, respectively, consistent with emergency conditions [4]. Although the World Health Organization (WHO) recommends integrating malnutrition in disease surveillance in the African region, there was no system to confirm and monitor the suspected nutritional crisis in the region [5].

Retrospective surveys can be used to monitor mortality in emergencies; however, unless they are repeated periodically, these surveys cannot provide information on changes over time [6]; furthermore, both household census and previous birth history approaches to mortality estimation require unfeasibly large sample sizes to achieve a precision sufficient to detect changes over relatively short periods (e.g., three months or even greater frequency of estimation in highly dynamic emergency scenarios). Community surveillance, while generally more expensive and labor-intensive than surveys, is a recognized approach to generate ongoing health data to capture trends [7]. This method is theoretically less biased than surveys as it is less affected by recall and information biases, and the prospective approach allows for quality to be improved over time [8,9]. Prospective surveillance provides for real-time monitoring and early warning of deteriorations, so as to inform immediate action by health service providers. Similarly, it allows health providers to detect substantial ameliorations in health conditions that may guide the decision to scale back programs. It may also promote long-term improvements in vital events recording, and if data management and analysis are automated, could in some scenarios be handed over to local health agencies with minimal researcher input. However, evidence is missing on whether such systems can reliably be implemented in crisis settings and particularly in rural, dispersed communities.

We set up a nutritional and mortality surveillance system in four sous-préfectures in the southwestern region of CAR, where Médecins Sans Frontières (MSF) implemented pediatric and nutritional activities, to monitor trends in death rates, acute malnutrition prevalence, and coverage of nutritional treatment.

Six months after its implementation, we evaluated the system to assess its performance.

## Methods

### Study population

**Figure 1:**
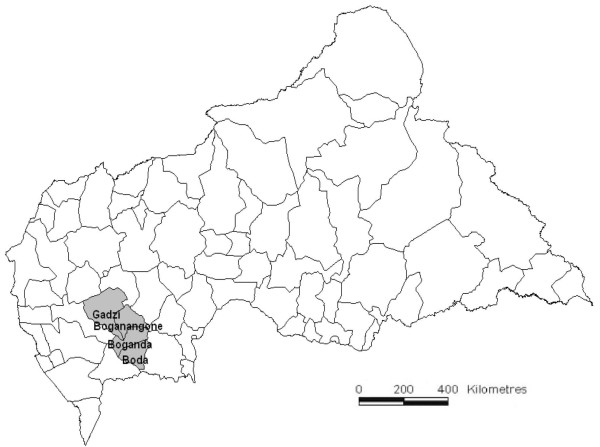
Map of the Central African Republic with location of the sous-préfectures in which surveillance was implemented.

CAR is divided into 16 prefectures that are further subdivided into 71 sous-préfectures. The study was conducted in Boda, Boganangone, and Boganda sous-préfectures (part of Lobaye prefecture) located in the Plateaux region and Gadzi sous-préfecture (part of Mambéré-Kadéï prefecture) located in the Equateur region (Figure 1). This is a mostly rural, forested area, with hundreds of small settlements located along unpaved roads or paths, scattered over an area of 14,993 square kilometers. Driving distances across the area are on the order of six to 10 hours. The area was estimated based on census projections to host a population of 158,000, of whom 46% (72,000) were children under 15 years old. The surveillance system was based at the community level and implemented in a random selection of sentinel sites within the area under surveillance.

### Indicators and sample size considerations

The system was designed to estimate prospectively the indicators listed in Table 1, over the entire area under surveillance, which we divided into a sampling frame of sites (villages or urban *quartiers* [neighborhoods]). Based on an estimated 450 people per site, selecting 24 sentinel sites from this sampling frame would have yielded a total sample under surveillance of about 10,000 people (including 1,600 children under 5 years [11]) or 85 households (groups of people living together and sharing meals) per site (assuming a mean household size of 4.9 [11]). We estimated the demographic indicators (death rates, birth rates) exhaustively, i.e., by collecting data on a weekly basis from each household within the sentinel sites. We estimated nutritional indicators based on a weekly systematic sample of all eligible children in every sixth household within each sentinel site, or about 340 children per week assuming one eligible child per household. Assuming a design effect of 1.5, the above samples enabled estimation of a monthly crude death rate (CDR) of 0.5 per 10,000 person-days with precision ±0.3, a monthly under-5 death rate (U5DR) of 1.0 per 10,000 child-days with precision ±1.0, a weekly global acute malnutrition (GAM) prevalence of 25% with precision ±8%, and a weekly severe acute malnutrition (SAM) prevalence of 5% with precision ±4%.

**Table 1:** Main indicators under surveillance

**Indicator**	**Explanation**	**Notes**
**Crude death rate**	Number of deaths due to all causes per 10,000 person-days	Also expressed as deaths per 1,000 person-years
**Under-5 death rate**	Number of deaths due to all causes among children under 5 per 10,00 child-days	As above
**Prevalence of global acute malnutrition (GAM)**	Percentage of children aged 6 to 59 months with middle upper arm circumference (MUAC) <125 mm and/or bilateral edema, defined as sustained pitting on dorsum of both feet after three seconds of thumb pressure	In practice, children were classified as falling within the target age range based on a proxy height criterion (65 to 110 cm). Note that this MUAC-based prevalence cannot be compared directly with more commonly used weight-for-height-based prevalence estimates [10]
**Prevalence of severe acute malnutrition (SAM)**	Percentage of children aged 6 to 59 months with MUAC <115 mm and/or bilateral edema	As above
**Coverage of SAM treatment**	Percentage of children with SAM that receive treatment for malnutrition by the MSF-supported program	The denominator includes those children normally resident in the sentinel site that may be temporarily admitted to inpatient facilities for complications
**Coverage of pediatric treatment**	Percentage of all children under 15 who died, whose caregivers reported a history of treatment in MSF-supported inpatient facilities during their final illness	Proxy indicator of the true coverage

### Selection of sentinel sites

We divided the sampling universe into six strata (Table 2). Within each stratum, we updated the 2003 census sampling frame of sites by touring the main road axes, reviewing health maps produced by health centers, and interviewing key informants. We grouped small, neighboring villages into clusters of about 500 people based on rough population figures. In order to obtain a sample representative of people’s proximity to health services, we sorted the sampling frame of sites (*quartiers*, villages, or clusters of villages) first by driving distance from the nearest health facility and second by urban versus rural. From this sampling frame, we selected by random systematic sampling four sentinel sites per stratum for a total of 24 sites (six strata with four sites each). All consenting households in each sentinel site were then enrolled in the system.

**Table 2:** Timeline of surveillance activities

**Dates (2010)**	**Weeks (2010)**	**Activities**
January 20 to February 1	3 to 4	Preparation:
		▪ Cartography and listing of sites to prepare sampling frame
		▪ Division into strata and selection of sentinel sites
		▪ Development and adaptation of procedures and registers
February 2 to March 7	5 to 9	Stepwise implementation (about two strata per week):
		▪ Identification of staff
		▪ Visit to sentinel sites
		▪ Three-day training and pilot
		▪ Baseline census during first week, then weekly household visits for demographic and nutritional data collection
March 8	10	Weekly household visits started in all sentinel sites
July 12 to 18	28	Repeat census
October 3	39	End of nutritional data collection
October 3 to 14	39 to 40	Evaluation visit
		▪ Capture-recapture study for mortality accuracy assessment (analyzed deaths during weeks 31 to 38)
		▪ Review of paper registers
December 31	52	End of demographic data collection

Four sites were unsuitable (three had <10 households; one was unreachable) and were thus replaced with the geographically closest suitable alternative. Note that this sampling strategy results in a self-weighting sample of sites within each stratum and, critically, does not require knowledge of accurate population figures, unlike for a cluster survey.

### Data collection

#### Implementation

Implementation activities started on January 20, 2010 and proceeded stratum by stratum in a stepwise approach, with the last site commencing data collection on March 8, 2010 (Table 2).

After obtaining the approval of civil authorities of each sous-préfecture and site and ethics approval from the Ethics Review Board of MSF, we trained one literate home visitor per site and three field supervisors over three days. In each site, a map with clear boundaries was drawn and five sectors (one per working day of the week) defined. Field questionnaires and consent instruments were translated into Songho from French and back-translated to ensure consistency with group consensus on final translations. A one day pilot data collection exercise was held, and standard operating procedures were written (available from the authors).

#### Demographic data collection

During the inception visit, home visitors sought verbal consent from household heads, or, if absent after three visits, the most senior household member aged ≥18. In consenting households, they then performed a baseline census, asking respondents to count all people who had slept in the household during the previous night but excluding extended family members and permanent emigrants.

Thereafter, on a weekly basis, proceeding sector by sector in a roughly consistent order, home visitors visited each household in each sentinel site, recording deaths, births, and in- and out-migration since the previous visit. Information on the treatment history of decedents was also sought. Each week, home visitors also verified whether new households had arrived or constituted themselves in the site; these were offered inclusion in the system as above. Similarly, home visitors identified households that had left the site or disintegrated and collected information on their composition from neighbors.

As data collectors did not track the number of people that "aged into" and "aged out of" age strata, with time the denominator for the U5DR became inflated (since births were added but children turning 5 years old were not subtracted). For this reason, and in order to evaluate the accuracy of weekly data collection on population movements, a repeat census was carried out in all sites 20 weeks after system implementation.

#### Nutritional data collection

During their weekly visits, home visitors stopped at every sixth household to collect anthropometric and nutritional program coverage data from all eligible children within the household. Home visitors did not select households in which to collect nutritional data. Each week, the sampling step (every sixth household) remained constant, but the first household to sample rotated from one to six on the list. To ensure this, week-specific registers were provided to the home visitors, indicating households in which to do anthropometry. This sampling process meant that the same children were assessed every six weeks.

Within each household sampled and for each child meeting the age (6 to 59 months old) or height (65 to 110 centimeters) criterion, home visitors measured the middle-upper arm circumference (MUAC), assessed the presence of bipedal edema (as a case definition of kwashiorkor malnutrition), and verified whether the child carried a bracelet denoting current enrolment in the MSF-supported nutritional program. They referred any children with MUAC <115 millimeters and/or edema and without a bracelet to the nearest MSF facility. MUAC and edema were used to classify nutritional status, as they are easy to measure by home visitors working alone in the community, enable quick on the spot classification of the child’s malnutrition status, and are more predictive of the risk of dying than weight-for-height indices [10].

#### Data management and analysis

Supervisors checked registers submitted by home visitors for abnormal or missing values and discussed these with home visitors during the following visit. They then single-entered data on EpiData masks featuring various range and consistency checks. Data were analyzed in the field using a software application we developed ad hoc (Meerkat), written in R language [12]. Meerkat captures Epidata databases, cleans and analyzes data, and generates an automatic Microsoft Word report (details and programming codes can be obtained from the authors).

We estimated indicators for the entire sampling universe using generalized linear models (assuming a Poisson distribution for demographic rates, offset by the natural log of the population in each site at the start of each analysis period, a Binomial distribution for proportions, and a normal distribution for means). Standard errors were adjusted for clustering of variables within sites, and various sampling weights were introduced to account for unequal site selection probabilities across the strata. A comparison of the baseline and repeat census with population estimates obtained through weekly tracking of in- and out-migrations suggested considerable bias in the weekly data collection (see Results). For this reason, for demographic rates we relied on census data as the denominator (i.e., a fixed population in each site during weeks 10 to 27 equal to the baseline census and a second fixed value during weeks 29 to 52 equal to that from the repeat census).

We report here data collected starting in week 10 (March 8, 2010), when all sentinel sites were up and running. For mortality, we report data up to week 52 (ending on December 31, 2010). For nutritional status and coverage, we report data up to week 39 (ending on October 3, 2010), as nutritional assessment was discontinued after this date in parallel with MSF scaling down operations.

### Monitoring and evaluation of the surveillance system

Unannounced supervision visits were carried out in all sites by supervisors with an approximately monthly or bimonthly frequency to verify correct implementation of data collection procedures and retrain home visitors if needed. Approximately six months after implementation we carried out a formal evaluation, as follows.

#### Accuracy of mortality estimates

To estimate the accuracy of the system for detecting deaths, we carried out a capture-recapture study using an approach similar to Roberts et al. [13]. Briefly, we generated three alternative lists of deaths occurring over the same time period (weeks 31 to 38, i.e., approximately the two months preceding the evaluation visit) among households within 16 of the 26 sites (the remaining 10 were inaccessible due to the rainy season). The first list consisted of deaths recorded by our surveillance system during weeks 31 to 38. The other two lists were created by consulting, respectively, birth attendants and religious leaders in each site, who were interviewed separately and in the absence of the home visitor and site authorities. We asked each of these key informants to list any households that had experienced a death over the same two-month period of interest. For each death reported by either key informant and/or the surveillance system, the team visited the household and, if verbal consent was provided, confirmed that a death did indeed occur and carefully verified the date of death through a detailed events calendar to make sure that it fell within the two-month period of interest.

Deaths on each list were cross-matched through unique identifiers (family name, age, gender, village of residence, date of death), and the sensitivity of the system’s mortality estimates was estimated using capture-recapture analysis as in Roberts et al. [13].

Separately, we also computed the positive predictive value of the mortality estimates: the denominator consisted of the total number of deaths recorded by the system over the two-month evaluation period, and the numerator consisted of all deaths that were recorded by the system and were subsequently confirmed as genuine during household visits for the capture-recapture analysis.

#### Accuracy of anthropometric measures

Throughout system implementation, we tallied (a) the number of children classified as SAM and referred by home visitors to MSF-supported outpatient nutritional facilities; (b) those among these children who actually were seen at these facilities; and (c) those among these children who met SAM criteria according to repeat anthropometry performed by MSF staff (assumed to be more reliable than the home visitors).

The above data enabled us to quantify the proportion of children that accessed nutritional care after referral (b/a) as a further proxy of intervention coverage and the positive predictive value of home visitors’ SAM classification (c/b).

#### Other evaluation activities

We reviewed a randomly selected sample of 35/744 (4.7%) weekly registers by comparing paper with electronic data. We also compared the repeat census data for each site with the population of each site, as determined by weekly updates to the baseline census (i.e., the summation of births, deaths, and migration in and out of the site up to the repeat census).

## Results

### System output

The 24 sentinel sites contained 3,969 households at baseline. Strata appeared relatively similar (Table 3), with the exception of Gadzi-Mbali commune stratum, where communities were more populous. In Boganda stratum, one site reported an unusually high mean household size (10.2).

**Table 3:** Profile of sentinel sites for each stratum and overall based on baseline census findings

**Stratum**	**Sites in sampling frame**	**Sentinel sites selected**	**Median number of households per site (range)**	**Median population per site (range)†**	**Mean household size (range)‡**
**Boda - Boda commune**	58	4	106 (66–182)	528 (377–854)	5.2 (4.7-6.1)
**Boda - Ngotto commune**	29	4	165 (88–179)	585 (383–862)	4.5 (4.0-5.3)
**Boganda**	29	4	141 (129–149)	783 (505–1287)	6.2 (3.9-10.2)
**Boganangone**	40	4	131 (112–362)	456 (420–1904)	5.1 (4.3-5.5)
**Gadzi - Mbali commune**	78	4	240 (182–250)	1046 (841–1132)	4.5 (4.3-4.6)
**Gadzi - Topia commune**	87	4	173 (113–239)	665 (428–962)	4.3 (3.4-5.1)
**Total (Four sous-préfectures)**	321	24	156 (66–362)	695 (377–1904)	4.9 (3.4-10.2)

† Includes consenting households only. ‡ Computed based on consenting households only.

At baseline, 26/3,969 (0.7%, range: 0.0% to 3.6% depending on the site) of households refused to take part in the surveillance system, and 252/3,969 (6.3%, range: 0.0% to 27.0%) could not be contacted. Thus, 3,691/3,969 (93.0%) of households were included in the system.

A mean of 3,898 households were visited every week, for a total of 163,718 household visits; on 18 of these visits (0.01%) the household opted out of the system, while on 6,774 (4.1%) visits, no adult household representative could be contacted to carry out the interview.

### Demographic structure of the population

At baseline, participating households contained a population of 18,081, consisting of 9,164 females (50.7%), 3,600 (19.9%) children under 5, 5,117 (28.3%) children aged 5 to 14, 7,307 (40.4%) people aged 15 to 44, and 2,057 (11.4%) people 45 and older.

Over the period of analysis, the crude birth rate was 61.8 (95% confidence interval [CI]: 54.7-69.9) per 1,000 person-years, ranging from 23.0 to 182.9 depending on the site.

### Mortality trends

**Figure 2:**
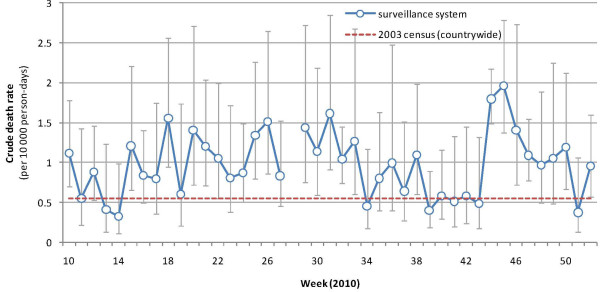
**Crude death rate by week, 2010. Vertical bars represent 95% CIs.** The crude death rate estimated for CAR as a whole in the 2003 census is also shown as reference.

The CDR was 1.0 (95% CI: 0.8-1.2) deaths per 10,000 person-days (35.0 deaths per 1,000 person-years) over the analysis period, ranging from 0.3 to 2.1 deaths per 10,000 person-days depending on the site. There was no obvious trend (Figure 2), but the highest values were observed during the rainy season (May to September 2010) and in November 2010. The CDR was 1.5 to three times higher than the 2003 census countrywide estimate [11].

**Figure 3:**
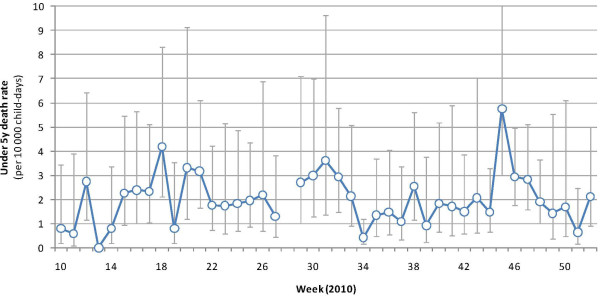
**Under-5 death rate by week, 2010.** Vertical bars represent 95% CIs.

The U5DR was 2.0 (95% CI: 1.5-2.6) deaths per 10,000 child-days over the analysis period (73.0 deaths per 1,000 child-years). Trends in under-5 death rates were similar, though the November peak was more marked (Figure 3).

The majority of deaths (286/510 or 56.1%) occurred among males (Table 4). About 40% of deaths were among children under 5; of these, one-third were neonatal and about half occurred in the first year of life.

**Table 4:** Distribution of deaths by age group and gender

**Age**	**Males**		**Females**		**Total**	
	**n**	**%**	**n**	**%**	**N**	**%**
**<5 years**	111	38.8	100	44.6	211	41.4
<1 month†	35	31.5	31	31.0	66	31.3
1 to 11 months†	29	26.1	17	17.0	46	21.8
1 to 4 years†	47	42.3	52	52.0	99	46.9
**5 to 14 years**	31	10.8	18	8.0	49	9.6
**15 to 44 years**	67	23.4	48	21.4	115	22.5
**≥45 years**	77	26.9	58	25.9	135	26.5
**Total**	**286**	**100.0**	**224**	**100.0**	**510**	**100.0**

† Percent of all deaths among children under 5.

### Nutritional status

Over the entire analysis period, 16,104 sets of anthropometric measures were collected and analyzable. The prevalences of GAM and SAM were 11.9% (95% CI: 9.1-15.5) and 3.0% (95% CI: 2.3-4.0), respectively. Of 484 children with SAM, 224 (46.3%) were reported to have edema, giving a prevalence of kwashiorkor of 1.3% (95% CI: 0.9-1.9); of these, however, only 53/224 (23.7%) were also classified as SAM by the MUAC <115 millimeters criterion.

**Figure 4:**
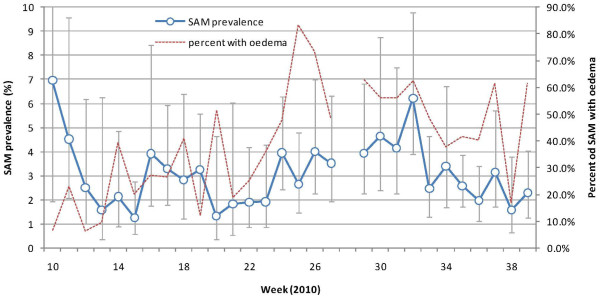
Prevalence of SAM (according to MUAC and/or edema; vertical bars represent 95% CIs) and percentage of SAM cases with edema, by week, 2010.

From a very high baseline of 7% (possibly reflecting inaccuracy during system implementation), SAM prevalence appeared to peak in April to May 2010, and more markedly in June to October, falling steadily thereafter (Figure 4). Notably, the proportion of SAM cases with edema was around 20% up to May, but thereafter increased to around 50%.

The mean MUAC value among children sampled showed relatively little variation during the entire period, averaging 141 millimeters (trends data not shown).

### Program coverage

**Figure 5:**
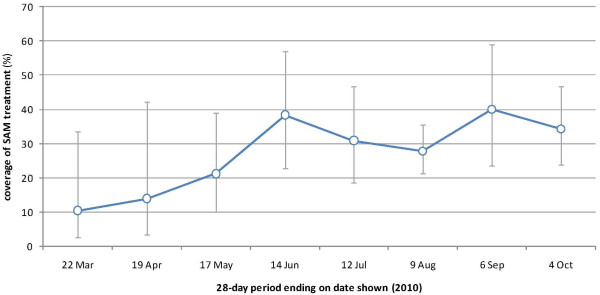
Coverage of SAM by month, 2010.

Between weeks 10 and 39, 129/489 or 29.1% (95% CI: 20.0-38.2) children with SAM had an MSF program bracelet (i.e., were under treatment). There were no statistically significant differences in SAM coverage across strata, though the lowest values were noted in Boganda (13.9%) and Boganangone (19.1%) sites. SAM coverage increased during 2010, from about 10% to 15% initially to 35% to 40% (Figure 5).

An alternative measure of coverage is provided by follow-up of referrals made by surveillance site home visitors. Of 599 children 65 to 110 centimeters tall (6 to 59 months old) classified as SAM cases by home visitors and referred to the MSF nutritional program (note that this denominator includes some who were not part of the weekly sample), only 134 (22.4%) were eventually seen by an MSF clinic.

Lastly, of 510 decedents of all ages during the analysis period, 140 or 28.5% (95% CI: 18.7-38.4) were reported by household respondents to have received some form of biomedical health care during their final illness. Among 260 children under 15 years who died, 67 or 30.2% (95% CI: 13.7-46.8) received care in MSF-supported health facilities.

### System evaluation

#### Accuracy of mortality estimates

Out of all deaths listed by key informants in the capture-recapture study, two could not be confirmed as the household had moved away, while 17 were excluded from the analysis after household visits (two were stillbirths, seven occurred outside the recall period, and eight decedents did not reside on site).

Similarly, out of 45 deaths listed by the surveillance system, six were excluded (two were among nonresidents, two were outside the recall period though within the period following the start of the surveillance system, and two were stillbirths). Based on these data (and accepting the two deaths outside the recall period as valid), the positive predictive value (PPV) of mortality reporting by the system was 41/45 (91.1%).

**Figure 6:**
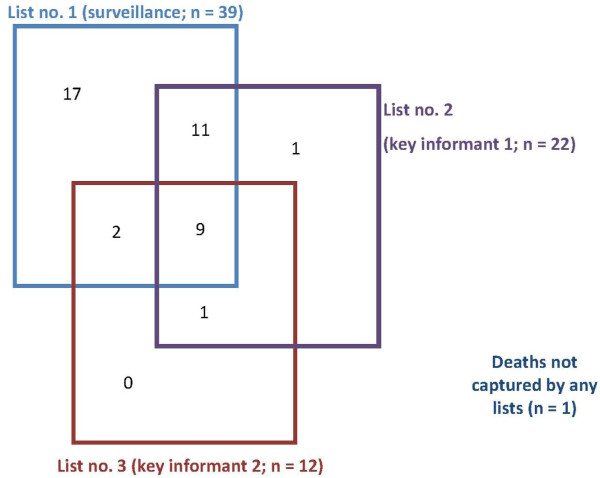
Venn diagram of the overlap in deaths reported among the three lists used for capture-recapture analysis.

The overlap among the three lists is shown in Figure 6. The surveillance system identified 39/41 deaths reported on any list. Capture-recapture analysis estimated that only one death was not detected by any list, yielding a system sensitivity of 39/42 (92.9%), greater than that of religious leaders (52.4%) and birth attendants (28.6%). Of 21 deaths among children under 5, all were estimated to have been captured by the surveillance system (sensitivity 100.0%), whereas religious leaders and birth attendants had lower sensitivity (42.9% and 19.0%, respectively).

#### Accuracy of anthropometric measures

Of 395 children sampled who met criteria for referral, 349 were referred (sensitivity of referral = 88.4%). An additional 56 were referred out of 18,383 who did not meet the criteria (specificity = 99.7%). Thus, of 405 referrals, 86.2% were warranted, assuming no error in MUAC and edema measurement by home visitors.

However, of 134 children referred with SAM by the surveillance system and seen by the MSF nutritional program, only 68 (50.7%) were confirmed as SAM cases according to MUAC (<115 mm), weight-for-height Z score deviation from the reference mean (<3 Z scores), and/or presence of bilateral edema. Adjusting for the proportion of unwarranted referrals among children sent to the program (about 14%), the PPV of the surveillance system's case definition of SAM was thus roughly 60%, using MSF nutrition staff's repeat measure as the gold standard.

#### Completeness and stability

Data were collected for 1,003/1,008 (99.5%) site-weeks. Two out of 35 weekly paper registers showed discrepancies with the electronic database. No interruption of data collection was reported, with the exception of week 28 (repeat census).

#### Accuracy of population denominators

**Figure 7:**
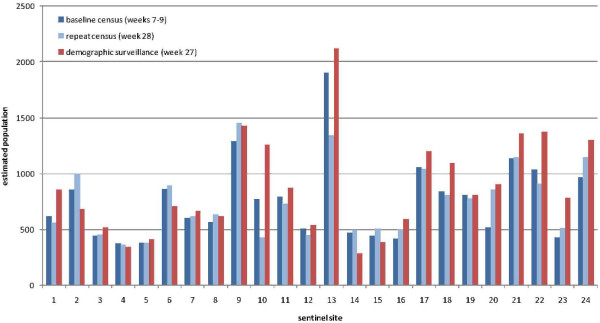
Comparison of baseline census (weeks 6 to 9, 2010), repeat census (week 28, 2010), and demographic surveillance (week 27, 2010) population estimates, by site.

**Figure 8:**
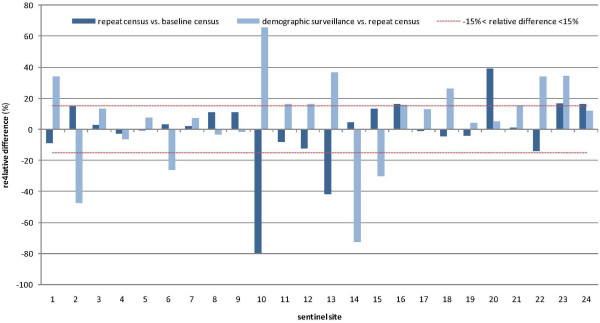
Relative difference (as percent) between baseline census, repeat census, and demographic surveillance, by site.

Population estimates by site according to the baseline census (weeks 7 to 9, 2010), repeat census (week 28), and demographic surveillance (week 27) are shown in Figure 7. The two censuses yielded similar estimates (relative difference <15%) for 21/24 sites, while in three sites (10, 13, and 20) large differences were noted (Figure 8).

Demographic surveillance yielded strikingly different population estimates in several sites, with relative differences >15% compared to the repeat census in 10/24 sites, and a higher estimate than the census in 17/24 sites. Demographic surveillance estimates were consistently higher than either census in the Gadzi sous-préfecture sites (sites 16 to 24).

Across all 24 sites, the population under surveillance was estimated to be 18,081 at baseline, 18,014 at the repeat census (−0.4%), and 21,099 (+16.7% from the baseline) according to demographic surveillance (sites 10 and 13 again appear problematic and account for much of this apparent increase). As of week 52, demographic surveillance reported 24,609 people under surveillance (+36.1% from the baseline).

## Discussion

We demonstrated that it was possible to implement community surveillance and validate it in a timely fashion in a crisis-affected setting featuring remote, scattered populations and scant demographic and geographical data. The system featured low attrition and appeared to be acceptable to the community, as demonstrated by the low percentage of absent households and the high response rate.

Demographic indicators such as household size and age structure were broadly consistent with national figures and other sources, suggesting the system produced robust and valid data at least during censuses (Table 5). However, we found a significantly and implausibly higher birth rate compared to the 2003 national census and other sources. Low contraceptive use, response bias and a high level of fertility in the area or period studied could all have played a role to inflate the crude birth rate; there could also have been a high proportion of women of fertile age in this area, but this seems unlikely, as the age-gender structure was reasonably close to the national one.

**Table 5:** Comparison of surveillance findings with other sources of demographic estimates

**Source (years of data collection)**	**Mean household size**	**Age distribution (%)**	**Crude birth rate (per 1,000 per year)**	**Crude death rate(per 1,000 per year)**	**Infant mortality as percent of under-5 deaths (%)**
DHS (1994–1995) [14]	4.9	<5 years: 16.7; 5 to 14 years: 30.2; 15 to 44 years: 38.9; ≥45 years: 11.1	-	-	61.4
MICS (2000) [15]	6.6	<5 years: 16.3	-	-	67.5
Census (2003) [11]	4.9	<5 years: 16.1	39	20	60.0
MICS (2006) [16]	4.6	<5 years: ≈17	-	-	60.2
Epicentre (2009) [4], Carnot sous-préfecture	5.3 (rural) 5.8 (urban)	<5 years: 21.3 (rural), 22.0 (urban)	-	27 (rural) 43 (urban)	-
Columbia University (2009) [17], South stratum	6.8	<5 years: 18.3	61	90	-
Vinck & Pham (2009) [18], Lobaye region	7.3	-	-	47	-
US Census Bureau projections (2010) [19]	-	<5 years: 15.6; 5 to 14 years: 25.5; 15 to 44 years: 44.9; ≥45 years: 14.0	37	15	67.5
**Our surveillance system**	**4.9**	**<5 years: 19.9; 5 to 14 years: 28.3; 15 to 44 years: 40.4; ≥45 years: 11.4**	**62**	**35**	**53.1**

### Mortality

Surveillance estimates of crude death rates were higher than in the 2003 census but similar to those from a survey in neighbouring Carnot and a larger study done in the Lobaye region (Table 5). These rates are approximately double those expected in stable sub-Saharan African settings [20] and indicate sustained excess mortality in this large population throughout 2010, consistent with a protracted health crisis.

Although the system did not collect information on causes of death, the age and sex distribution of deaths suggests a spectrum of diseases typical of sub-Saharan Africa. The higher mortality in males versus females may reflect greater, possibly occupational (e.g., mining) exposure to injury, HIV/AIDS, and tuberculosis. Neonatal and infant deaths are likely attributable to multifactorial events related to maternal health, inadequate prenatal care, low skilled birth attendance, short birth spacing, preterm birth, low birth weight, and infections as already observed elsewhere [21,22]. We also observed a peak in death rates during October and November 2010, possibly due to an infectious disease outbreak.

### Malnutrition

The prevalence of GAM at almost 12% was elevated, although we do not have a good basis against which to benchmark severity, as existing gravity thresholds rely on weight-for-height Z-score indices and not on MUAC. Previous analyses suggest that, with the exception of pastoralist populations (only a small minority in our area of CAR), MUAC and weight-for-height, while not capturing the same children, yield a broadly similar prevalence of acute malnutrition [23-25]. Some evidence of a seasonal peak of GAM in the second part of the year, coinciding with a higher proportion of kwashiorkor among SAM cases, was observed. The SAM prevalence (2% to 4%) is very concerning, particularly since kwashiorkor has a higher case-fatality ratio than marasmus [26,27].

Trends in treatment coverage were monitored as a key determinant of the MSF intervention's impact. Throughout 2010 we estimated SAM and pediatric treatment coverage well below 50%. However, an encouraging upward trend was noted for SAM coverage, suggesting steady improvement. Past outpatient SAM treatment programs have achieved coverage closer to 70% to 80%, but these were implemented in settings with easier access to the population [28]. Distance, community awareness of the program, and the way in which rejections are handled at program clinics are reported as the main reasons for incomplete coverage in outpatient nutritional care [29].

### System performance and limitations

Our evaluation allowed us to verify the accuracy of the system in estimating the indicators of interest. The system showed a high sensitivity and specificity for mortality (>90%) and good sensitivity and specificity for SAM. However, only 60% of children referred for SAM were actually confirmed as SAM by MSF teams, indicating a low PPV, possibly due to difficulties in detecting edema and insufficient training and monitoring of home visitors. We cannot in turn exclude misclassification by MSF nutrition staff (used as the gold standard for confirming SAM) that could have resulted in an artificially low PPV.

A key limitation of our system is that we did not perform verbal autopsies to ascertain likely causes of death. These should always be administered by a clinician and results interpreted by a panel of medical doctors, which would have been unfeasible to implement in CAR on a prospective basis due to the scarcity of health professionals (a recent method based on random forest statistics, however, appears to perform better than a clinician panel [30]). Furthermore, important ethical and logistics issues have been highlighted when verbal autopsies are included in routine surveillance [31] that we did not feel we could surmount given resources available. It could have been possible to couple a verbal autopsy study with the capture-recapture assessment, as done previously [32].

There is currently no solid evidence on the minimum number of clusters (or sentinel sites) that should be sampled to yield robust mortality estimates, though for anthropometry 20 to 25 appear sufficient [33]. While selecting a greater number of sites would have been preferable, in practice we had to balance statistical considerations with serious logistical and resource constraints.

A major problem with weekly tracking of population movements seems obvious. Data on in- and out-migration did not seem reliable and tended to inflate the denominator over time, resulting in a progressive underestimation of death rates. Had we used the weekly surveillance denominator instead of census estimates, the estimates of CDR, U5DR, and crude birth rate over the entire analysis period would have been 0.7 (95% CI: 0.6-0.9) per 10,000 person-days (compared to 1.0 using census data), 1.4 (95% CI: 1.0-1.8) per 10,000 person-days (compared to 2.0), and 47.9 (95% CI: 42.5-53.9) per 1,000 person-years (compared to 61.8). We were unable to identify reasons for this probable bias; incorrect understanding of procedures by home visitors (e.g., failure to exhaustively record people leaving households or households that ceased to exist) or misreporting (voluntary or involuntary) by households (e.g., short-term visitors may have been reported as additions to the household but not departures when they left) may be responsible. In general it is plausible that with time communities may become fatigued with weekly data collection and increasingly misreport information. In order to circumvent this problem in the future, we suggest regularly carrying out repeat censuses instead of weekly tracking of population movements. This approach will also adjust for aging into and out of different age groups.

Another limitation with sentinel site surveillance is that as a result of surveillance activities leading to referral of SAM cases, health status may improve within the sentinel sites, making these sites progressively less representative of the area under surveillance. Given the low SAM and pediatric treatment coverage observed, such bias is likely to be limited. In the future, however, we suggest rotation of sentinel sites every six to nine months: we expect that this arrangement would reduce the above measurement bias and prevent deteriorations in data quality due to possible loss of interest in routine data collection by communities or lack of ongoing training of home visitors.

## Conclusions

This study demonstrates that it is possible to establish valid prospective community surveillance to monitor demographic, mortality, nutritional, and coverage indicators providing robust data in a large, neglected crisis-affected area. The high but stable excess mortality and SAM prevalence suggest a chronic and silent crisis in this area of CAR. Crises in remote and scattered populations are becoming an increasingly frequent operational scenario for relief agencies [34]. Prospective surveillance systems similar to ours should increasingly be considered in such scenarios and can be modified to include additional relevant indicators, such as vaccination coverage or household water and sanitation access. Operationally, a focus on improving populations' access and increasing the coverage of curative interventions will be critical to achieving an appreciable health impact in such populations.

## Abbreviations

CAR: Central African Republic; CI: Confidence interval; GAM: Global acute malnutrition; MSF: Médecins Sans Frontières; MUAC: Middle upper arm circumference; SAM: Severe acute malnutrition.

## Competing interests

The authors declare that they have no competing interests.

## Authors' contributions

All authors contributed to study design. GMC, APS, JP, and FC implemented data collection. GMC, SB, and FC performed analysis and computer programming. All authors interpreted findings and contributed to writing this manuscript. All authors read and approved the final manuscript.
